# Overcoming time-varying confounding in self-controlled case series with active comparators: application and recommendations

**DOI:** 10.1093/aje/kwae216

**Published:** 2024-07-19

**Authors:** Anna Schultze, Jeremy Brown, John Logie, Marianne Cunnington, Gema Requena, Iain A Gillespie, Stephen J W Evans, Ian Douglas, Nicholas Galwey

**Affiliations:** Department of Non-communicable Disease Epidemiology, London School of Hygiene and Tropical Medicine, London WC1E 7HT, United Kingdom; Department of Non-communicable Disease Epidemiology, London School of Hygiene and Tropical Medicine, London WC1E 7HT, United Kingdom; Department of Epidemiology, Harvard T.H. Chan School of Public Health, Boston, MA 02115, United States; GlaxoSmithKline PLC, London WC1A 1DG, United Kingdom; GlaxoSmithKline PLC, London WC1A 1DG, United Kingdom; Analysis Group, London EC2R 7HJ, United Kingdom; GlaxoSmithKline PLC, London WC1A 1DG, United Kingdom; GlaxoSmithKline PLC, London WC1A 1DG, United Kingdom; Department of Non-communicable Disease Epidemiology, London School of Hygiene and Tropical Medicine, London WC1E 7HT, United Kingdom; Department of Non-communicable Disease Epidemiology, London School of Hygiene and Tropical Medicine, London WC1E 7HT, United Kingdom; GlaxoSmithKline PLC, London WC1A 1DG, United Kingdom

**Keywords:** self-controlled case series, unmeasured confounding, fluoroquinolones, thiazolidinediones, Clinical Practice Research Datalink

## Abstract

Confounding by indication is a key challenge for pharmacoepidemiologists. Although self-controlled study designs address time-invariant confounding, indications sometimes vary over time. For example, infection might act as a time-varying confounder in a study of antibiotics and uveitis, because it is time-limited and a direct cause of both receipt of antibiotics and uveitis. Methods for incorporating active comparators in self-controlled studies to address such time-varying confounding by indication have only recently been developed. In this paper, we formalize these methods and provide a detailed description for how the active comparator rate ratio can be derived in a self-controlled case series: either by explicitly comparing the regression coefficients for a drug of interest and an active comparator under certain circumstances using a simple ratio approach or through the use of a nested regression model. The approaches are compared in 2 case studies, one examining the association between thiazolidinedione use and fractures and one examining the association between fluoroquinolone use and uveitis, using the United Kingdom’s Clinical Practice Research Datalink. Finally, we provide recommendations for the use of these methods, which we hope will support the design, execution, and interpretation of self-controlled case series using active comparators and thereby increase the robustness of pharmacoepidemiologic studies.

**This article is part of a Special Collection on Pharmacoepidemiology**.

## Introduction

The self-controlled case series (SCCS) is a “self-matched” study design, in which the risk of an outcome during exposed and unexposed time periods is compared within cases who experience the outcome^1^^,^^2^. A particular strength of the SCCS is that both measured and unmeasured time-invariant confounding factors are inherently controlled for through the design, which has benefits when studying causal questions potentially subject to unmeasured confounding. Although the method was developed for studying adverse events after vaccination,^1^ it has also been applied to the study of other drug safety questions and the impact of environmental exposures on health outcomes.^2^

The SCCS design has some important limitations: It relies on a number of potentially restrictive assumptions.^3^ Briefly, these state that outcomes should be independently recurrent or rare, that the occurrence of the event should not affect the future probability of exposure, and that the occurrence of the event should not affect the observation time. The SCCS is also susceptible to time-varying confounding, which can limit the validity of findings.^2^ While age and calendar-time effects can be easily incorporated, accounting more generally for time-varying confounders can be challenging, as they may not be measured or can be challenging to adjust for despite being measured. For example, indications for treatments are often unmeasured, which can cause concern around unmeasured confounding when these change over time. One approach to controlling for such time-varying confounding is the use of active comparators: this reduces the scope for bias due to unmeasured time-varying confounders, since a well-chosen active comparator is expected to have a similar time-varying pattern of confounding.^4^ Active comparators are commonly used in cohort and case–control studies in pharmacoepidemiology to address confounding by indication,^5^ but they are less frequently applied to SCCS, since they are not permitted within the basic method. Methods for their incorporation in these settings have only recently been developed,^4^ and there are few examples of their application.

Our aim in this paper is to describe and compare different methods for the incorporation of active comparators in the SCCS design. We first set out the methods formally, and we then evaluate them in 2 studies where time-varying confounding by indication was a concern, using data from the United Kingdom’s Clinical Practice Research Datalink (CPRD). The first study examined the association between thiazolidinedione use and fractures^6^ and the second the association between fluoroquinolone use and uveitis.^7^ Finally, we discuss considerations for the incorporation of active comparators in SCCS and present a series of recommendations for researchers looking to use these methods.

## Methods

### The SCCS study design

The SCCS design assumes that events relating to an individual arise from a nonhomogeneous Poisson process. For each individual $i$*,* the follow-up time is split into risk and reference periods defined by a binary exposure, with risk periods denoted $X=1$ (eg, 1-60 days following each prescription) and reference periods denoted $X=0$ (eg, all other time). The standard SCCS model of the event rate, ${\mathrm{\lambda}}_{ix}$, for outcome $Y$ is then


**Figure 1:**
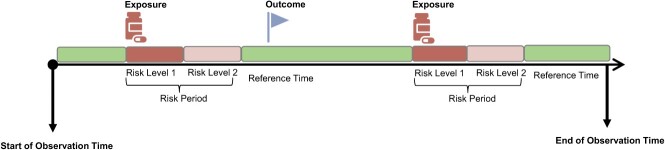
Illustration of a standard self-controlled case series study design.

(1) \begin{equation*} \mathbb{E}\left[Y|I=i,\kern0.5em X=x\right]={\mathrm{\lambda}}_{ix}={\mathrm{\phi}}_i\exp \left(\mathrm{\beta} x\right), \end{equation*}

where $\mathrm{\beta}$ is the relative effect of the exposure on a logarithmic scale and ${\mathrm{\phi}}_i$ represents the effect of time-invariant individual characteristics on the event rate. During the analysis the likelihood is conditioned on the number of events ${n}_i$ occurring over the entire observation time in individual *i*, and this results in ${\mathrm{\phi}}_i$ being eliminated from the likelihood. This explains why the method controls for time-invariant confounding. Note that ${n}_i$ does not relate to a single risk period, and conditioning on it therefore does not compromise the comparison between different periods within the same individual. The method can easily incorporate multiple categorical risk periods (eg, days 1-30 after first prescribing, days 31-60, etc), as illustrated in Figure 1. In a simple scenario where the risk period has just one level, the quantity of interest in an SCCS is the rate ratio (RR):


(2) \begin{equation*} \frac{\phi_i\exp \left(\mathrm{\beta} \right)\ }{\phi_i\exp (0)} = \exp \left(\mathrm{\beta} \right). \end{equation*}

The RR contrasts the within-individual frequency of an event occurring during a risk period to the frequency of it occurring during unexposed reference time (typically, all time less the risk period) among those who experience the event. Each case may experience multiple risk periods, and each risk period can be further classified into multiple levels representing different hypothetical levels of risk^8^ (Figure 1).

### Active comparators

The purpose of incorporating an active comparator in an SCCS is to compare the rate for the treatment of interest with the rate for the comparator treatment. Two potential reasons for this are (1) to assess and account for the effect of time-varying confounding by indication and (2) to compare the causal effect of 2 different treatments. In some circumstances, especially for adverse events, we may assume that the active comparator has no causal effect on the outcome, but by including it we may be able to reduce time-varying confounding by indication.^9^

We can derive the active comparator RR in an SCCS in several different ways. Firstly, we can calculate a ratio of RRs by estimating the effect of each drug on the outcome, dividing the estimated ratios, and then calculating a CI (the simple ratio approach).^4^ As is discussed further in the next section, under certain assumptions this ratio of ratios is equivalent to the active comparator RR. Alternatively, the equivalent quantity can be derived in a single step using a regression model that can be specified in a variety of ways (the “nested model” approach).

### The simple ratio approach

One approach to estimating the active comparator RR is to calculate a ratio of ratios. Let ${\mathrm{\beta}}_1$ represent the parameter (log RR) for the effect of a risk period caused by exposure to the drug of interest, and let ${\mathrm{\beta}}_2$ represent the corresponding parameter for the comparator drug. The estimator is then given by


(3) \begin{equation*} \frac{\exp \left({\mathrm{\beta}}_1\right)\ }{\exp \left({\mathrm{\beta}}_2\right)} = \exp \left({\mathrm{\beta}}_1-{\mathrm{\beta}}_2\right). \end{equation*}

While this is a simple approach, it is not immediately clear what the underlying estimand is or whether this matches up with the desired estimand (ie, the active comparator RR). When the two comparator treatments (*X* and *Z*) are mutually exclusive or nearly mutually exclusive and the baseline reference time is unexposed to either treatment, the ratio of ratios does simplify to an active comparator RR:


(4) \begin{equation*} \frac{E\left[Y|I=i,X=1,Z=0\right]}{E\left[Y|I=i,X=0,Z=0\right]}/\frac{E\left[Y|I=i,X=0,Z=1\right]}{E\left[Y|I=i,X=0,Z=0\right]}=\frac{E\left[Y|I=i,X=1,Z=0\right]}{E\left[Y|I=i,X=0,Z=1\right]}, \end{equation*}

where *X* is a binary variable representing a risk period caused by exposure to the drug of interest (unexposed: *X* = 0; exposed: *X* = 1) and *Z* is a binary variable representing a risk period caused by exposure to the comparator. Alternatively, when there is substantial overlap in treatments periods, if there is either no interaction between treatments or we include an interaction term ${\mathrm{\beta}}_3$ in the model, the ratio of ratios (equation (3)) simplifies to the active comparator RR (equation (4)).

The simple ratio method, as originally described by Hallas et al,^4^ is based on estimating the effect for each drug in 2 separate case series. Alternatively, estimates for the effect of the drug of interest and comparator on the outcome can also be derived by fitting a single regression model and including both exposures:


(5) \begin{equation*} \mathbb{E}\left[Y|I=i,\kern0.5em X=x,Z=z\right]={\mathrm{\phi}}_i\exp \left({\mathrm{\beta}}_1x+{\mathrm{\beta}}_2z\right). \end{equation*}

One benefit of deriving the estimates for $\exp (\widehat{\beta_1}$) $\exp (\widehat{\beta_2}$) from a single model is that we can easily obtain the covariance between these estimates from the fitted model, which then contributes to the calculation of the 95% CI for the active comparator RR. Formulas for these calculations are provided in Appendix S1. A further benefit of including more cases in one model is increased precision when adjusting for calendar time or age effects, assuming common age/calendar-time temporal effects.^10^

Multiple levels of risk are easily accommodated in the simple ratio method, by splitting the risk period caused by exposure to each drug into different periods based on potentially different levels of risk, represented by categorical variables. For example, a preexposure period and 2 postexposure risk periods might result in a categorical exposure variable with the following levels: 0 (reference time), 1 (preexposure), 2 (days 1-30), and 3 (days >30). These can be included as a series of dummy variables for each risk level, here designated using subscripts:


(6) \begin{align*} &\mathbb{E}\left[Y|I=i,\kern0.5em {X}_1={x}_1,{X}_2={x}_2,\kern0.5em {X}_3={x}_3,\kern0.5em {Z}_1={z}_1,\kern0.5em {Z}_2={z}_2,\kern0.5em {Z}_3={z}_3\right]\nonumber\\&={\mathrm{\phi}}_i\exp \left({\mathrm{\beta}}_1{x}_1+{\mathrm{\beta}}_2{x}_2+{\mathrm{\beta}}_3{x}_3+{\mathrm{\beta}}_4{z}_1+{\mathrm{\beta}}_5{z}_2+{\mathrm{\beta}}_6{z}_3\right). \end{align*}

We can then apply the above principles to each risk level to derive the active comparator incidence ratio for that level. For example, the active comparator RR for the preexposure period becomes


(7) \begin{equation*} \frac{\exp \left({\mathrm{\beta}}_1\right)\ }{\exp \left({\mathrm{\beta}}_4\right)} = \exp \left({\mathrm{\beta}}_1-{\mathrm{\beta}}_4\right). \end{equation*}

### Nested model

An alternative derivation of the active comparator incidence ratio involves fitting a regression model with nested exposure variables. For this purpose we define a new variable, *E*, representing a risk period caused by exposure to either the drug of interest or the comparator (*X* = 1 or *Z* = 1). As before, *X* represents a risk period caused by exposure to the drug of interest. The active comparator RR can then be derived by fitting the following model:


(8) \begin{equation*} \mathbb{E}\left[Y|I=i,\kern0.5em X=x,\kern0.5em E=e\right]={\mathrm{\phi}}_i\exp \left({\mathrm{\beta}}_1e+{\mathrm{\beta}}_2 xe\right). \end{equation*}

If the two treatments are mutually exclusive, then ${\mathrm{\beta}}_2$ in this model represents the active comparator RR, whereas ${\mathrm{\beta}}_1$ represents the incidence ratio for the comparator of interest compared with no exposure, among those not exposed to the drug of interest. Although this parameterization has been referred to as an interaction approach,^4^ expressing the model in the standard statistical-model notation of Wilkinson and Rogers^11^ makes it clear that this model represents a special case where *X* is nested in *E* (Appendix S2). We have therefore chosen to refer to this as the “nested model” approach. When the risk period is represented by a binary variable, it can be shown that the nested model can be further simplified:


(9) \begin{equation*} \mathbb{E}\left[Y|I=i,\kern0.5em X=x,\kern0.5em E=e\right]={\mathrm{\phi}}_i\exp \left({\mathrm{\beta}}_1e+{\mathrm{\beta}}_2x\right). \end{equation*}

This is because *X* is equivalent to *X* × *E* when *X* takes only the values 1 and 0. Because these models are equivalent, which one is fitted is a matter of user preference. One challenge with the nested model approach, which is not shared by the simple ratio approach, is that if the risk periods associated with each drug are not mutually exclusive, ${\mathrm{\beta}}_3$ will not estimate the ratio of the rate with the drug of interest alone to the the rate with comparator alone, but rather the ratio of the rate with the drug of interest with or without the comparator to the comparator drug alone.

The nested model approach can also be extended to incorporate multiple risk levels. This can be done by extending *E* to a categorical variable. Using the same risk levels as above, this variable would then take on the following values: 0 (reference time), 1 (preexposure period for *X* or *Z*), 2 (days 1-30 for *X* or *Z*), and 3 (days >30 for *X* or *Z*). *X* would still take on the value 1 when *E* represented a risk period caused by exposure to the drug of interest, and 0 otherwise. Equivalently, dummy variables for each risk level can be created manually, and the setup in equation (8) replicated for each level.

There is another way of deriving the active comparator RR that can be used when an individual can only be exposed to either the drug of interest or the comparator but not both, as might be the case for some vaccines. In this situation, we can create a case series with a variable denoting ever exposure to either the comparator or the drug of interest during a person’s observation time. The value of this variable is constant for each person, which allows the active comparator incidence ratio to be derived through an interaction term (Appendix S2). Although relatively simple to conceptualize, this method is likely to have relatively limited applicability, as it requires that patients have only a single type of exposure throughout the observation period; therefore it will not be considered further.

### Notes on implementation

Researchers implementing the method should carefully consider how they will handle potentially overlapping risk periods between the drug of interest and the comparator. If risk periods are not mutually exclusive but are nevertheless counted as contributing only towards the risk period for the drug of interest, ${\mathrm{\beta}}_2$ will represent the active comparator RR only if it is assumed that there is no direct effect of the comparator drug on the outcome (conditional on being exposed to the drug of interest). To avoid making this assumption with the nested model approach, overlapping time periods can be treated as a separate level in a multilevel categorical exposure variable. This will fit a model equivalent to including an interaction term between risk periods caused by exposure to each drug in the single regression model simple ratio approach. Whether this is worthwhile will depend on the extent of overlap between risk periods. When there are several risk levels (eg, days 0-30, days ≥30), including an interaction term between treatments in the simple ratio approach, or adding separate levels for joint treatment in the nested model, can lead to sparse strata and issues in model convergence. Generic Stata code for implementing both the simple ratio and nested approaches is available on GitHub (https://github.com/annaschultze/active-comparator-sccs).

## Results from case studies

To evaluate the methodology, we applied and compared the simple ratio and nested model approaches in 2 studies using data from CPRD GOLD and Aurum, 2 databases of anonymized primary-care records from the United Kingdom.

### Thiazolidinediones and fractures

Thiazolidinediones are antidiabetic agents used to treat type 2 diabetes. There has been historical concern that their use may increase the risk of fractures. Using an SCCS design, Douglas et al^6^ investigated this question in CPRD GOLD and found that thiazolidinedione use was associated with an increased risk of experiencing fractures after adjustment for age (RR = 1.43; 95% CI, 1.25-1.62). To investigate whether residual time-varying confounding by progression of diabetes could potentially explain the observed association, the study was repeated among patients prescribed another class of antidiabetic agents: sulfonylureas. Prescribing of these drugs was not associated with an increased fracture risk (RR = 0.84; 95% CI, 0.66-1.08).

We repeated the analyses by Douglas et al to incorporate sulfonylureas formally as an active comparator. Analyses were based on 2 already-created case series in which all required variables were present, and the simple ratio method was therefore implemented using 2 separate case series. To allow us to evaluate the nested models, we applied an additional censoring requirement, in which we censored individuals at treatment discontinuation. This allowed us to stack the datasets into a single dataset with consistent specifications. The study had only a single risk level, so all exposure variables were binary.

We included 1089 individuals, 885 exposed to thiazolidinediones and 213 exposed to sulfonylureas. Results from the separate case series are presented in Table 1. Use of thiazolidinediones was associated with an increased risk of fracture, with the association attenuated but still significant upon adjustment for age in 1-year age bands. Sulfonylurea use was not associated with an increased risk of fractures.

**Table 1:** Associations of thiazolidinedione use and sulfonylurea use with fracture risk.^a^

**Model**	**Drug class and RR (95% CI)**	
	**Thiazolidinediones**	**Sulfonylureas**
Unadjusted	2.23 (1.93-2.58)	1.08 (0.74-1.58)
Adjusted for age	1.50 (1.25-1.80)	0.70 (0.47-1.05)

Abbreviations: CPRD, Clinical Practice Research Datalink; RR, rate ratio.

^a^Data were obtained from CPRD GOLD (1987-2007).

Results from formally incorporating the active comparator, before and after adjustment, are presented in Table 2. All analyses found an increased risk of fractures, and agreement between the simple ratio and nested model approaches was good in unadjusted analyses. After age adjustment, the nested model approach resulted in a somewhat lower estimate than the simple ratio approach.

**Table 2:** Results from an active comparator analysis of the association between thiazolidinedione use and fracture risk.^a^

**Model**	**ACRR (95% CI)**	
	**Simple ratio**	**Nested model**
Unadjusted	2.06 (1.37-3.11)	2.06 (1.37-3.11)
Adjusted for age	2.14 (1.37-3.33)	1.88 (1.23-2.87)

Abbreviations: ACRR, active comparator rate ratio; CPRD, Clinical Practice Research Datalink.

^a^Data were obtained from CPRD GOLD (1987-2007).

In this study, formally incorporating sulfonylurea use as an active comparator slightly increased the strength of the observed association between thiazolidinedione use and fracture risk, although 95% CIs remained wide. This highlights the value in presenting the results from individual case series when incorporating an active comparator, as any apparent harm observed for the drug of interest may be driven by a protective effect of the comparator. The point estimates from the simple ratio and the nested model, though identical in unadjusted models, differed slightly upon adjustment for age. This slight difference was due to 2 separate case series’ being used in the simple ratio approach but only a single one, including patients exposed to both drugs, for the nested model approach. This meant that adjustment for age in the nested model approach was based on a common estimate of the appropriate coefficient in a broader population pool, whereas adjustment in the simple ratio approach was based on separate estimates in the two case series.

### Fluoroquinolones and uveitis

Fluoroquinolones have been associated with potential safety concerns, including uveitis and collagen-associated events such as tendon rupture.^12^ It is not clear to what extent the associations reported between fluoroquinolones and uveitis are causal, since the infection which led to treatment with these antibiotics may also increase the risk of uveitis. Brown et al^13^ conducted a cohort study and an SCCS study, using both CPRD GOLD and Aurum, to investigate the association between fluoroquinolone use and acute uveitis. Because there was concern about time-varying confounding by indication, cephalosporins, a group of antibiotics used for similar indications, was added as an active comparator.

As an addition to the original analyses, we extended the analyses by applying the nested approach to incorporating active comparators, and compared this with the simple ratio approach. The study used 3 different risk levels (days 1-29 from initial exposure, days 30-59, and days ≥60), incorporated using a series of dummy variables (binary indicator variables, taking the value 1 for person-time in the relevant risk period and 0 otherwise). A 30-day preexposure period was added to account for potential violations of one of the core underlying assumptions of the SCCS, namely that the occurrence of the outcome does not affect the probability of exposure. A preexposure period can mitigate some violations of this assumption, when the effect of the outcome on exposure probability is short-lived. We anticipated this to be the case here, as uveitis may decrease primary-care prescribing in the short term (due to hospitalization).

Day of prescribing (day 0) was categorized as separate levels. We did not consider repeat occurrences of uveitis; that is, only the first occurrence of uveitis within the study period was considered an outcome event. However, given that uveitis is a rare event, this was anticipated to introduce minimal bias.^14^ A single Poisson model was fitted without interaction terms between exposures, separately in GOLD and Aurum, in both the simple and nested approaches.

We included 72 251 incident cases of acute uveitis identified in Aurum, of whom 12 947 patients were exposed to fluoroquinolones and 18 111 were exposed to cephalosporins. From GOLD we included 8301 incident acute uveitis cases, of whom 1436 were exposed to fluoroquinolones and 1909 to cephalosporins. Results from separate analysis for each drug, adjusted for age and calendar time, are presented in Table 3. Briefly, there was weak evidence of an association between fluoroquinolone use and uveitis at days 1-29 and 30-59. The RRs were closer to 1 at days ≥60, although 95% CIs were wide in that latter time period. There was also weak evidence of an association between cephalosporins, the control antibiotic, and uveitis at days 1-29, although the estimates of association moved towards the null in later time periods.

**Table 3:** Associations^a^ of fluoroquinolone use and cephalosporin use with uveitis.^b^

**Risk window, d**	**Drug class and RR (95% CI)**	
	**Fluoroquinolones**	**Cephalosporins**
1-29	1.13 (0.97-1.31)	1.16 (1.04-1.30)
30-59	1.16 (1.00-1.34)	1.03 (0.92-1.16)
60-end	0.98 (0.74-1.31)	0.87 (0.70-1.09)

Abbreviations: CPRD, Clinical Practice Research Datalink; RR, rate ratio.

^a^The model adjusted for age and calendar time.

^b^Data were obtained from CPRD GOLD (1987-2019) and Aurum, 2 databases of anonymized primary-care records from the United Kingdom.

Results from formally incorporating cephalosporins as a comparator are presented in Table 4. Using the simple ratio method resulted in null results for days 1-29 and weak to no evidence of an association in later time periods. The simple ratio and nested models gave very similar results.

**Table 4:** Results from an active comparator analysis^a^ of the association between fluoroquinolone use and uveitis.^b^

**Risk window, d**	**ACRR (95% CI)**	
	**Simple ratio**	**Nested model**
1-29	0.97 (0.81-1.17)	0.97 (0.81-1.17)
30-59	1.13 (0.94-1.36)	1.14 (0.94-1.37)
60-end	1.13 (0.79-1.63)	1.14 (0.79-1.63)

Abbreviations: ACRR, active comparator rate ratio; CPRD, Clinical Practice Research Datalink.

^a^The model adjusted for age and calendar time.

^b^Data were obtained from CPRD GOLD (1987-2019) and Aurum (1995-2019), 2 databases of anonymized primary-care records from the United Kingdom.

In this second case study, incorporating cephalosporins as an active comparator resulted in estimated incidence ratios for the association between fluoroquinolones and uveitis that were closer to the null for days 1-59 than those from the analyses without the comparator. The weak association observed between cephalosporin use and uveitis was unexpected and has not been widely reported in the literature. Such an association could be explained by confounding by indication, as infection is a risk factor for uveitis and thereby can act as a time-varying confounder. Thus, incorporating the comparator formally in this instance looks to have removed a small potential increase in the RR driven by time-varying confounding by indication.

## Discussion

### Considerations and recommendations

The simple ratio and nested regression model methods will give similar results in most circumstances, but there may be specific scenarios where a particular method is preferable. The simple ratio method is somewhat more flexible than the nested model approach, since fitting 2 separate case series in principle allows for different sets of adjustment variables for the drug of interest and the comparator.^4^ However, it is difficult to conceive of a situation where a time-varying confounder would be important to adjust for one of the drugs but not the other, as the comparator drug is likely to be chosen specifically because it shares a similar confounding structure to the drug of interest. The simple ratio method also allows for the incorporation of overlapping risk periods between the two drugs in quite a straightforward manner. However, if the simple ratio approach is implemented using 2 separate case series, the method assumes that there is zero covariance between the two drugs for the purposes of constructing CIs. This may not be reasonable in all circumstances and could lead to differences in the coverage if the covariance is large. As we have demonstrated, fitting a single regression model including effects of both the drug of interest and the comparator can overcome these problems.

Differences may also be introduced between the simple ratio and nested model methods if the simple ratio estimates are derived from 2 separate case series, as the study populations contributing to covariate adjustments would then differ. This could also be mitigated by fitting both the simple ratio and nested models in one combined series, which would also offer the additional advantage of allowing potentially better control of any age- or time-related confounding through more precise estimates of such effects (provided that the magnitude of the effects does not differ strongly between the two case series). The inclusion of unexposed cases to allow for better control of age- or time-related confounding in SCCS has been previously suggested,^10^ and the differences between the simple ratio and nested models found here also illustrate the value of this approach when such confounding is strong.

It has been recommended to implement more than one method and compare the results^4^; however, it may be preferable to choose one method as a primary analysis strategy based on the specific requirements of a certain question and implement the others as sensitivity analyses given their theoretical equivalence. Whichever method is chosen, we recommend that the RR for the association between both drugs and the outcome should be presented to allow the researchers to assess whether their assumptions concerning the direction of bias due to unmeasured time-varying confounding were accurate.

## Conclusions

The active comparator methodology was introduced for several different self-controlled study designs in a 2021 paper by Hallas et al.^4^ This covered not only the SCCS but also the case-crossover design, case-time-control studies, and sequency symmetry analyses. In this paper we have focused on the SCCS, to enable an in-depth exploration of the methods associated with this design, but many of the considerations we raise here will apply when active comparators are incorporated in other self-controlled study designs as well. Note that our first case study found no strong evidence of association between the comparator drug and the outcome, and the difference between the standard and active-comparator RRs was therefore relatively small. However, applying an active comparator was still valuable in this scenario, since it provided reassurance that the association between thiazolidinediones and fractures was unlikely to be due to confounding by indication. The proposed methods are relatively new and have still not been that widely applied. Chui et al^15^ applied an active comparator in an SCCS study looking at the association between proton pump inhibitors (PPI) and myocardial infarction, using H2 receptor antagonists as active comparators. They used the simple ratio method, and were able to estimate an active comparator ratio for PPIs corrected for substantial time-varying confounding by indication. Taking this correction into account, they recovered a null effect between PPIs and MI.^15^

We hope that our detailed exploration of these methodologies will be a guide for other researchers interested in applying active comparators in their own studies, and thereby increase the uptake of these methods.

## Supplementary Material

Web_Material_kwae216

## Data Availability

The data used in these analyses are protected by patient confidentiality rules and cannot be shared. Researchers can apply to access CPRD data at https://www.cprd.com/data-access.
