# Supplementary material for: Overcoming time-varying confounding in self-controlled case series with active comparators: application and recommendations
Source: Am J Epidemiol. 2024 Jul 19;194(1):220–5. doi: 10.1093/aje/kwae216 (PMC11735952; doi:10.1093/aje/kwae216)
Supplement: Web_Material_kwae216 [file web_material_kwae216.pdf]

SUPPLEMENTARY MATERIAL

Overcoming Time-Varying Confounding in Self-Controlled Case Series with Active Comparators: Application and Recommendations

Anna Schultze, Jeremy Brown, John Logie, Marianne Cunnington, Gema Requena, Iain A. Gillespie, Stephen J. W. Evans, Ian Douglas, and Nicholas Galwey

Table of Contents

Appendix S1. Formulas for 95% CI for the active comparator rate ratio using the simple ratio approach 2

Appendix S2. Wilkinson and Roger notation 3

Appendix S1. Formulas for 95% CI for the active comparator rate ratio using the simple ratio approach

A confidence interval for the active comparator rate ratio can be constructed by summing the variance for $\exp(\widehat{\beta_1})$, $\exp(\widehat{\beta_2})$ and two times their covariance:

$$\mathrm{var}(\widehat{\beta_1} - \widehat{\beta_2}) = \mathrm{var}(\widehat{\beta_1}) + \mathrm{var}(\widehat{\beta_2}) + 2 * \mathrm{covar}(\widehat{\beta_1}, \widehat{\beta_2}) \quad (1)$$

The square root of the variance for the log active comparator rate ratio is the standard error, and this can be used to construct Wald confidence intervals:

$$\mathrm{se}(\widehat{\beta_1} - \widehat{\beta_2}) = \sqrt{\mathrm{var}(\widehat{\beta_1} - \widehat{\beta_2})} \quad (2)$$

$$\exp((\widehat{\beta_1} - \widehat{\beta_2})) \pm 1.96 * \mathrm{se}(\widehat{\beta_1} - \widehat{\beta_2})) \quad (3)$$

Note that the calculations are done on the natural log scale. If it is not possible to use a single model to derive the covariance, a confidence interval can still be derived if it can be assumed that there is zero covariance between the two coefficients representing risk periods caused by exposure to the drugs.

Appendix S2. Wilkinson and Roger notation

Let X represent a risk period caused by exposure to the drug of interest: unexposed, X = 0; exposed, X1 = 1. Likewise, let Z represent a risk period caused by exposure to the comparator drug, and E a risk period caused by exposure to either the drug of interest or the comparator. That is, if X = 1 *or* Z = 1 then E = 1. As specified in the current paper, the active comparator incidence ratio can then be derived by fitting the following model with X nested within E, and without explicitly specifying Z:

$$\mathbb{E}[Y|I = i,\ X = x,\ E = e] = \phi_i \exp\,(\beta_1 e + \ \beta_2 ex) \quad (1)$$

This can be clarified by expressing the model in Wilkinson and Rogers notation. Using this notation it becomes clear that the effects of E and X are not crossed:

$$Response \sim E * X \quad (2)$$

This specification would call for the estimation of the main effect of both E and X. Instead, these variables are nested:

$$Response \sim E/X \quad (3)$$

In other words, the effect of X is only estimated when E = 1.
